# Regulation of GMO field trials in the EU and new genomic techniques: will the planned reform facilitate experimenting with gene-edited plants?

**DOI:** 10.5114/bta.2023.125086

**Published:** 2023-03-27

**Authors:** Tomasz Zimny

**Affiliations:** Institute of Law Studies, Polish Academy of Sciences, Warszawa, Poland

**Keywords:** GMO, experimental release, field trial, new genomic techniques, risk assessment

## Abstract

This study presents the possible consequences of maintaining the current regulatory regime of the experimental release of genetically modified higher plants in the EU for the products of new genomic techniques (NGTs). Currently, the experimental release is a crucial stage before the authorization of a product for the market. By analyzing the data on the performance of field trials in the EU (numbers, sizes, dominating countries) and comparing the present regulatory provisions with those of selected third countries (including new provisions adopted in the UK), this study shows that the current framework of GMO (genetically modified organisms) field trials is ill-fitted for breeding activities. Due to strict limitations placed on the operator of a field trial in the EU, easing the regulatory burdens on the authorization of certain NGT products for the market may not provide researchers (especially, plant breeders) the competitive position they need if the present legal conditions for carrying out GMO field trials with certain NGT products (especially, those that are considered GMOs covered by the EU GMO legislation) are not going to change as well.

## Introduction

The European Commission (EC) plans to amend the EU GMO legislation so that it becomes more suited for the regulation of products of new genomic techniques (NGTs). In a study published in 2021 (European Commission, 2021), the EC defined NGTs as an umbrella term denoting various methods of gene editing that were profoundly developed in the past 20 years. ‘NGT’ refers to multiple techniques of interventions in the plant genome that have been developed since 2001. This is applicable to plants with single nucleotide variants, plants developed through the application of site directed nucleases (SDN)-1–2 or oligonucleotide directed mutagenesis (ODM) techniques, products of cisgenesis, and plants with stable inserts of large fragments of foreign DNA, such as products of SDN-3 techniques. In the same study, the EC observed that the present GMO regulatory framework may no longer be adequate to regulate the access of such products to the market and reported that the restrictions placed on such products should be proportional to risks associated with the genetic changes introduced in the plant. A broader application of such products could improve the competitiveness of agriculture in the EU and contribute to the success of the Green Deal and Farm to Fork strategies (European Commission, 2020). Multiple propositions regarding how the legislation should be amended have been published in recent years (Breyer et al., 2009; Bratlie et al., 2019; Eriksson et al., 2020b, 2020a; for an overview, see also Zimny and Eriksson, 2020), yet the actual contents of the EC’s proposal and its final shape are not known. What the EC has made clear so far is that the provisions regarding the market authorization of some NGT products should be relaxed to the extent that they still comply with laws that guarantee a high level of environmental and health safety. The outcome of this process would depend on the types of products that would be selected for the relaxation of provisions and on the proposed solutions that will find political support, if any. It is worth noting that although most of the discussions around the planned amendments to the EU legislation seem to be based on the assumption that all products of NGTs are considered GMOs, this is not the only position, since some academics maintain that certain products (e.g., SDN-1 products) should not be considered GMOs as they do not meet the legal definition of a GMO, because they can also be obtained through conventional methods (Van Der Meer et al., 2021). Some researchers maintain that due to the similarity of certain NGT products to the products of conventional breeding, they should be exempted from the legislation (Vives-Vallés and Collonnier, 2020). However, the prevailing view, as expressed by the EC (European Commission, 2021), seems to be that regardless of the level of intervention into the genome, the products of targeted mutagenesis or cisgenesis should currently be treated as regulated GMOs, though the EC claims that the legislation should be changed so that the regulatory burdens should be proportional to the risks involved in the use of a particular organism (European Commission, 2021). The latter perspective is taken as a departure point for further reasonings within this manuscript.

The stakeholders in the discussion about the shape of the change tend to focus on substantive and procedural aspects of the authorization of NGT plants for the EU market (see, e.g., Zimny and Eriksson, 2020). Little attention is paid to the step that necessarily precedes the authorization – the experimental release of GMO, termed “release for purposes other than placing on the market” by the 2001/18/EC Directive on the release of GMOs into the environment (Directive 2001/18/EC), which is usually carried out by conducting GMO field trials in the case of plants. In the recent survey conducted by the EC on various options that could be adopted in the framework of new legislation, GMO field trials were not considered, thus limiting an opportunity to address this issue only to an open text box, where the respondent could voice any opinion. None of the survey questions specifically addressed the performance of GMO field trials. However, it seems that easing the planning and performance of GMO field trials with certain plants developed using NGTs is crucial to the subsequent development of products for market authorization. Should the EU successfully adopt new legislation on the authorization of such products, the potential of such technology may not be used to its full extent without changes in the legislation on the planning and performance of GMO field trials.

## Experimental release of GMO higher plants into the environment in the EU

According to a primary interpretation of the provisions of the 2001/18/EC Directive, especially after the Court of Justice of the European Union (CJEU) judgment in the “mutagenesis case” (CJEU C-528/16, 2018) and the passing of the EC study on the legal status of some NGT products (European Commission, 2021), products of techniques such as directed mutagenesis, cisgenesis, and intragenesis and organisms in which the genetic material is altered without changes in the nucleic acid sequence (due to epigenetic changes) have to be treated as regulated GMOs (European Commission, 2021, 19–22). This indicates that the provisions of the EU legislation not only regarding the authorization of food and feed products (Regulation 1829/2003/EC) and other products (e.g., sowing material for the purpose of cultivation to the market, Directive 2001/18/EC – henceforth “Directive”) but also regarding the experimental release into the environment apply to these products (Directive 2001/18/EC). The latter issue is the focal point of this study.

The experimental release of any GMOs, including higher plants (Angiospermae and Gymnospermae, according to the Directive 2001/18/EC), is harmonized across the EU through part B of the Directive. Although actual provisions governing the conditions for the release and its authorization are parts of local statutory laws, they need to comply with the provisions of the Directive, along with its annexes (primarily Annex II, *Principles for the environmental risk assessment*; and Annex III, listing information needed to be included in the notification filed by the applicant with a competent authority). Hence, though there may be differences between some member states of the EU regarding the formalities and practicalities of obtaining permission for the experimental release of GMOs, the common core lies within the Directive, which will be used as a reference point throughout this article.

As per Article 6.1 of the Directive, any applicant wishing to release GMOs into the environment for experimental purposes must notify the competent authority of the member state in which the release is planned. The notification needs to contain a technical dossier and environmental risk assessment (as indicated below) and is processed by the authority (e.g., a minister of environment), which needs to issue a decision in 90 days (extended by any period required to gather additional information from the applicant for public consultations, which can take up to 30 days). GMOs should not be released into the environment without the written permission of the competent authority. The experimental release of GMOs, according to ordinary procedures, requires consultation with the public, in compliance with the rules devised by the member states (Article 9). Article 7 of the Directive describes differentiated procedures that could be applied to particular GMOs in cases, when sufficient experience has been obtained in certain ecosystems. This provision allows for reduced requirements the information related to GMOs, the conditions of their release, their interactions with the environment, and the environmental risk assessment. To date, such differentiated procedures were not applied.

**Fig. 1:**
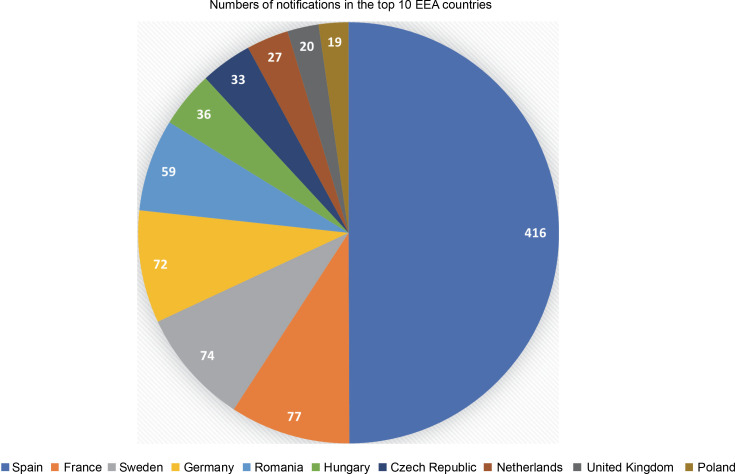
Number of GMO field trial notifications in the top 10 European Economic Area countries, filed between October 2002 and September 2022. (Source: European Commission, 2022; Zimny, 2022a)

It has been 20 years since the first notification of an experimental release of higher plants, based on the 2001/18/EC Directive, was filed (European Commission, 2022). Since then, the collective number of notifications has increased to 915, with 680 approved so far. Most of the notifications (416 or 45.5%) were filed in Spain, followed by France, Sweden, and Germany, accounting for around 8% each. Furthermore, the top ten (out of 31) European Economic Area (EEA) countries and the UK (which was included as it was part of the EU for most of the analyzed period) accounted for 833 (91%) of the total number of notifications (see Fig. 1).

The average number of notifications per year in the first decade of experimental releases (2002–2012) was about 81, whereas in the second decade (2013–2022), it was only about 10 (Zimny, 2022a). These data indicate that the number of experimental releases is not evenly distributed across the EEA, with researchers in multiple countries not filing even one notification, and also that the number of notifications has declined in the last decade. The rapid adoption of NGTs in plant research and breeding, the increasing number of peer-reviewed publications, and even Nobel Prizes (The Nobel Prize Organization, 2020, 2021) do not seem to affect the number of notifications. The reasons for the decrease in the number of GMO field trials are probably multifactorial, and their in-depth discussion is beyond the scope of this article. The negative perception of GMOs in the EU and the bureaucratic burdens associated with applying for and conducting a GMO field trial seem to be of importance (see, e.g., Woźniak-Gientka et al., 2022).

## Conditions of the experimental release and environmental risk assessment

### Conditions of the experimental release

The conditions for the experimental release of genetically modified (GM) higher plants (Angiospermae and Gymnospermae) were laid out in Annex III B, which was designed taking into account “classic” GMOs (i.e., featuring stable insertions of foreign DNA fragments). Hence, it requires the notifier (the entity applying for the permission) to provide information regarding the recipient or parental plants, the genetic modification, information related to the GM plant, information related to the site of release, release duration, control, monitoring, postrelease activities, waste treatment, etc. Looking at these requirements in detail allows one to notice that some are either not required in the case of NGT products featuring single nucleotide variants or even impossible to provide due to the nature of the change in the plant’s genome. This applies especially to the information regarding the insert or the vector, which might be impossible to provide in the case of plants featuring single nucleotide variants and information regarding the promoter, terminator regions, or probes. Furthermore, the provision of some of the information required will subsequently seriously limit the ability of the notifier to carry out conventional breeding practices, such as crossing, selection, and production of propagation material during field testing. These issues need to be discussed in detail.

Besides providing standard information regarding themselves, the notifier has to present data regarding the released organism. This part of the annex contains requirements that would significantly limit the ability to carry out breeding activities with the released organism developed by NGTs in a regulatory scenario, where such products would be considered GMOs that require authorization, even if such authorization were to be granted according to simplified procedures or less stringent criteria than in the case of “classic” GMOs. The notifier, *inter alia*, needs to provide information on the cultivar or the breeding line of the plant being released. This means that if the permission for the experimental release is subsequently issued, it is limited to the cultivar or the breeding line mentioned in the notification. Should any breeding activities carried out on the released plants result in obtaining a product that could be classified as a different cultivar or breeding line than the one mentioned in the notification – for instance, a population of plants with different conventional-like mutations obtained by NGTs – these plants would no longer be within the ramifications of the permission.

Part of the information required is either irrelevant or impossible to provide in the case of a notification of the release of an NGT product without a stable insert of a larger DNA fragment (e.g., resulting from the application of SDN-1–2 or ODM, featuring single nucleotide variants). This pertains primarily to the required information regarding the vector (Annex III B part C) or the insert and the vector (Annex III B part D.2).

The parts of the notification, and hence the permission to carry out an experimental release, which further limits its fitness (in the current state) to carry out plant breeding activities, are parts E and F of the annex, which regulate the required information about the release site and the release. The notifier is required to name the location of the planned release and describe the ecosystem, such as flora, fauna, presence of sexually compatible wild relatives or cultivated plant species, and proximity to officially recognized biotopes or protected areas potentially affected by the release. In addition, the authorities are to be provided with information about, *inter alia*, methods for preparing and managing the release site both during and after the release and the approximate number of plants or their density. In accordance with part G, the notifier is also required to list the precautions taken, such as distances from sexually compatible species, limitations of pollen, seed or tuber dispersion, and methods of monitoring or waste treatment.

In practice, the release is limited to a particular, relatively small field trial site. Of the 101 experiments notified in the past decade (2013–2022), a quarter had plot sizes smaller than 0.065 ha, whereas only 12 were outliers with areas higher than 2.9 ha. These data are further skewed by two huge experiments, one in Sweden and one in Spain, with the declared sizes of 1500 and 1839 ha, respectively. If these two data points are removed from the dataset, then all experiments bigger than 2.4 ha are counted as outliers (12) and the average plot size with those omitted is 0.4 ha (Zimny, 2022a). The scale of such experiments is thus limited, and though the current provisions do not place any upper limit on the size of the experimental site, the preparation of larger sites is usually associated with severer difficulties regarding the selection of the site, the separation of the experiment from sexually compatible plants grown in the vicinity of the site, and the establishment of physical barriers and other means used for risk limitation and management. These factors also limit the feasibility of the current GM experimental release regime to carry out plant breeding activities with NGT products. Until a product is authorized for the market, the production of propagation material through contracts with third parties is not possible, unless they were mentioned in the notification.

### Risk assessment

The principles for the environmental risk assessment of the GMOs released into the environment were defined in Annex II to the 2001/18/EC Directive. However, an in-depth discussion of these principles is beyond the scope of this article, and they are based on the same principles as at the time of the adoption of the Directive (Wilkinson et al., 2003). It is worth mentioning, however, that the environmental risk assessment serves one of the primary goals of the Directive – to protect human health and the environment while carrying out the experimental release of GMOs into the environment, in accordance with the precautionary principle (Art. 1). The principles of the environmental risk assessment involve the following: comparing the identified characteristics (which may cause adverse effects) of the released GMO with those of an unmodified counterpart (for further details, see Conner et al., 2003); carrying out a risk assessment based on scientific data in a sound and transparent manner; following a case-by-case approach; and reviewing the conclusions of the risk assessment, if new data become available (Annex II, part B).

It has been shown in the literature that certain risk assessment requirements for products of NGTs, especially those featuring single nucleotide variations (e.g., certain products of SDN-1 or SDN-2 techniques) and cisgenesis, may be either no longer applicable or simply not required in the safety assessment of such products. For instance, the European Food Safety Authority (EFSA) GMO panel concluded that the use of cisgenic plants may be associated with hazards similar to those of using conventionally bred plants, so risk assessment data for these plants could be reduced based on familiarity, on a case-by-case basis (EFSA Panel on GMO, 2012, 19–20). Similarly, the risk assessment requirements of SDN-1–2 or ODM products could also depend on these factors as the familiarity of the allele being edited (Naegeli et al., 2020, 8; see also Zimny, 2022b).

Regardless of the content of the provisions in the proposal for market authorization procedures for plants obtained through targeted mutagenesis or cisgenesis, risk assessment requirements for carrying out field trials also limit the access of EU researchers. Risk assessment needs to be carried out for particular plants planned to be released or particular cultivars mentioned in the application, if the planned experiment involves their use. As a change in the conditions of the release (e.g., introduction of cultivars not mentioned previously) requires an update and perhaps issuance of another permission or change in the conditions of the issued permission, the rigid framework of the current notification and permission system maintains a precautionary approach, at the cost of the flexibility of the conditions in which researchers are allowed to carry out their activities (for a more in-depth discussion on such approach, see Casacuberta and Puigdomènech, 2018). This scenario does not seem to be characteristic of the research and development activities in other countries, where agricultural products have been developed using NGTs described in this article.

## Provisions in selected other countries

The regulatory status of NGTs in third countries, which are large partners of the EU in the trade of GM agricultural products (Zimny and Sowa, 2021), differs from that endorsed by the authorities in the EU (Dederer and Hamburger, 2019; Eriksson et al., 2019; Zimny and Sowa, 2021); in particular, certain products of NGTs either do not qualify as regulated GMOs in those third countries or are – to a smaller or larger extent – exempted from their regulation. Certain NGT products could be either treated as regulated GMOs with lessened regulatory burdens in the proposed EU legislation or they could be exempted from the legislation, as the products of random mutagenesis currently are (Zimny, 2022b). The difference between the two aforementioned regulatory solutions (from the perspective of access to GMO field trials) is that if a certain type of plant is considered to be a regulated GMO, it should go through the official GMO field trial procedures. If the plant falls out of the regulatory regime, it is usually not subject to restrictions regarding GMO field trials as well, other than, e.g., an obligation to notify the competent authorities about the planned experiment so that they can review the regulatory status of the organisms planned to be released. The latter approach (the ability to carry out an experimental release in the absence of an objection of the competent authority) has been recently adopted in the UK.

### UK

The change of procedures to carrying out GMO field trials and marketing of gene-edited products in the UK seems to be one of the most striking ones introduced to the post-Brexit legislation. The government created a two-stage plan aimed at the facilitation of research and the subsequent introduction of certain gene-edited products to the market. The regulation on the deliberate release of GMOs (UK Parliament, 2022a) was amended in such a way that it exempts “qualifying higher plants” from the risk assessment obligation before the experimental release. Such a plant is defined as a “genetically modified organism but which has not been genetically modified other than to make modifications, that could have occurred naturally, or that could have been made using one or more of the techniques set out in regulation 5(2)” (generally through techniques excluded or exempted from the regulation, including mutagenesis). The technical guidance published by the Advisory Committee on Environmental Release (ACRE, 2022) affirms that plants with epigenetic changes, SDN-1 or SDN-2 products, and some cisgenic plants would likely be treated as “qualifying higher plants,” provided they meet the aforementioned definition. It is worth noting here that it is the nature of the change in the genome, rather than the technique that was used, that is decisive here. Under the provisions of the newly adopted regulation, individuals wishing to carry out the experimental release of “qualifying higher plants” are exempted from conducting a risk assessment and obtaining consent for the release and may carry out the release after a notification filed with the Secretary of State 20 days before the planned release. From a procedural point of view, this is a radical change in the situation for the researchers, and apparently, the first releases started about a month after the new regulations were adopted (Rothamsted Research, 2022).

### USA

The US system of authorization of GM products is governed by three agencies: the United States Department of Agriculture (USDA, which regulates certain GMOs as potential plant pests), the Food and Drug Administration, and the Environmental Protection Agency (when it comes to plant protection products, including those produced by modified plants). Recently, two major amendments to the system were made, under the name of the “Sustainable, Ecological, Consistent, Uniform, Responsible, Efficient” (SECURE rule) (USDA, 2020, now simply called “Revised Regulations”), which exempt certain plants from plant pest regulation. The exemptions apply to plants with a single modification of a type in one of three following categories: a change resulting from the cellular repair of a targeted DNA break in the absence of an externally provided repair template; a targeted single-base-pair substitution; and the introduction of a gene known to occur in the plant’s gene pool or a change in the targeted sequence to correspond to a known allele of such a gene or to a known structural variation present in the gene pool. Other plants may also be exempted based on previous knowledge regarding the plant trait mechanism of action (Hoffman, 2021).

From a procedural perspective, what distinguishes the US system from the others described in this article is that the former relies to a large extent on the compliance of entrepreneurs rather than on imposing mandatory participation in the authorization process. An operator may still choose to consult the relevant agency regarding the status of their product; however, should they decide not to, they would risk the consequences of not complying with the provisions of the relevant act.

### Argentina and Brazil

Both these countries have introduced changes to their GMO legislation that warrant a differentiation in the treatment of modified products, depending on the nature of the genetic change introduced in the genome. The Argentinian legislation defines a transformation event as “insertion in the plant genome in a stable and joint way, of one or more genes or DNA sequences that are part of a defined genetic construct” (Secretaria de Agricultura, Ganaderia, Pesca y Alim, 2011). According to literature sources, numerous cases of products of techniques such as SDN-1–2 and ODM were classified as non-GMOs, to which the regime created for conventional breeding products applies (Whelan and Lema, 2019, 26–29).

The Brazilian legislation uses the criterion of new genetic combinations being stably present in the product as a feature that distinguishes the product, which should go through the GMO authorization procedure, from the excluded products (Ministério da Ciência, Tecnologia, Inovações e Comunicações, 2018). The products that do not meet the regulated GMO definition (e.g., featuring single nucleotide variants) do not fall under the GMO regulatory regime.

The outcome of the adoption of such a regulatory solution is that breeders wishing to use techniques that legally do not lead to the creation of regulated GMOs are subjected to fewer restrictions, having to comply primarily with the requirements applicable to the products of conventional breeding.

## Conclusions

As of now, it is difficult to predict the aspects of the legislative proposal the EC is going to present or – more importantly – what changes are going to be adopted after the EU legislative process, if any (for further information, see Zimny, 2022b). However, it should be emphasized that the current policy discussions mostly pertain to the legal status and authorization procedures for certain NGT products entering the EU market. From a researcher’s perspective, the legal conditions for the testing, development, and breeding of improved plant varieties are equally important. If the existing regulatory regime to carry out GMO field trials remains as it is and all products of NGTs will be considered regulated GMOs [even with a less stringent regulation (for a different interpretation, see Van Der Meer et al., 2021)], then researchers and plant breeders in the EU will face bigger obstacles than their third-country counterparts. As the data presented above show, currently GMO field trials in the EU are limited not only in numbers but also in scale, and this is likely, to a large extent, due to the legal conditions of the experimental release. The limitations that researchers will face for an NGT product legally considered to be a regulated GMO (as opposed to their third-country counterpart, whose material is not considered either a GMO at all or a regulated GMO) include, but are not limited to:

- a requirement to receive permission for an experimental release,
- a requirement to provide exhaustive data to the competent national authority,
- a requirement to carry out a risk assessment for every planned experiment,
- inability to change the conditions of the experiment during the trial,
- inability to use third-party services to produce sowing material prior to the authorization, and
- being limited to the chosen sites and conditions of the approved experimental release.

As the aforementioned European Food Safety Authority opinions suggest, some of these requirements may not be necessary or even not possible to be met in the case of certain NGT products, especially those that could have otherwise been obtained using conventional breeding methods or conventional mutagenesis. If the EC succeeds in introducing provisions that allow for easier access of certain NGT products to the EU market, while still treating such products as regulated GMOs requiring authorization and maintaining the current regime of GMO field trials unchanged, then EU breeders are going to be at a disadvantage. This will apply to a situation where a given NGT product is considered a regulated GMO, which falls within the scope of the GMO legislation, as opposed to a situation where such a product would be exempted from the legislation altogether. Should the EC decide to introduce less stringent procedures for certain NGT products, while still maintaining their status as regulated GMOs, it should also propose changes in the legislation regulating the conduct of GMO field trials using such plants. Otherwise, entrepreneurs in third countries could develop ready-formarket products at a faster pace due to higher flexibility in devising field trials and carrying out breeding activities. At the same time, their EU counterparts will be limited to carrying out initial works in the rigid GMO field trial regime and can start large-scale breeding activities practically after having the product authorized for the market, which will delay the development of the final product by several years, taking into account the time devoted to carrying out the initial GMO field trial and authorization procedure, even if a shortened one. Inevitably, this will also lead to an increased product development cost. These problems would not apply to products that would be considered non-GMOs or that would be exempted from legislation, as has been suggested by some previous studies [e.g., for SDN-1 products (Vives-Vallés and Collonnier, 2020; Van Der Meer et al., 2021)]. Whether such solutions will be proposed or adopted is currently doubtful. However, it needs to be emphasized that without a change in the framework of GMO field trials for certain NGT plants, a reform encompassing the relaxation of the regulatory burdens for certain regulated products may turn out to be more beneficial for thirdcountry breeders than for those in the EU.

## Conflict of interest

The author declares no conflict of interest associated with this work.
